# Use of platelet concentrate gel in second-intention wound healing: a case report

**DOI:** 10.1186/s13256-020-02649-6

**Published:** 2021-02-18

**Authors:** Vincenzo Davide Palumbo, Stefano Rizzuto, Giuseppe Damiano, Salvatore Fazzotta, Andrea Gottardo, Giuseppina Mazzola, Attilio Ignazio Lo Monte

**Affiliations:** 1 Department of Surgical, Oncological and Oral Sciences, University of Palermo, Via Del Vespro, 129, 90127 Palermo, Italy; 2 Euro-Mediterranean Institute of Science and Technology (IEMEST), Palermo, Italy; 3 Department of Biomedicine, Neurosciences and Advanced Diagnostics, University of Palermo, Palermo, Italy

**Keywords:** Regenerative medicine, Skin flap, Surgery, Wound healing

## Abstract

**Background:**

Wound healing is a complex and dynamic process. Healing of acute and chronic wounds can be impaired by patient factors (that is, comorbidities) and/or wound factors (that is, infection). Regenerative medicine products, such as autologous/homologous platelet-rich plasma gel, may speed up the healing process. Autologous/homologous platelet-rich plasma is an advanced wound therapy used for hard-to-heal acute and chronic wounds. The cytokines and growth factors contained in platelet-rich plasma play a crucial role in the healing process.

**Case presentation:**

A 61-year-old Caucasian male patient, suffering from mental retardation following meningitis, with a transplanted kidney due to prior renal impairment, and under immunosuppressant therapy, was submitted to aneurysmectomy of his proximal left forearm arteriovenous fistula. A few days later, the patient came to our attention with substantial blood loss from the surgical site. The wound presented no signs of healing, and after fistula reparation and considering persistent infection of the surgical site (by methicillin-resistant *Staphylococcus aureus*), surgeons decided for second-intention healing. To favor healing, 10 mL homologous platelet concentrate gel was sequentially applied. After each application, wound was covered with nonadherent antiseptic dressing. After only seven applications of homologous platelet concentrate gel, wound completely recovered and no amputation was necessary.

**Conclusions:**

Topical application of homologous platelet-rich plasma gel in healing wound shows beneficial results in wound size reduction and induces granulation tissue formation. Platelet-rich plasma could be a safe and cost-effective treatment for managing the cutaneous wound healing process to shorten the recovery period and thereby improve patient quality of life.

## Background

Wound healing is a complex and dynamic process. Healing of acute and chronic wounds can become impaired by patient factors (that is, comorbidities) and/or wound factors (that is, infection). Regenerative medicine products, such as autologous/homologous platelet-rich plasma (PRP) gel, may speed up the healing process [1]. Autologous/homologous PRP is an advanced wound therapy used in hard-to-heal acute and chronic wounds. The cytokines and growth factors contained in PRP play a crucial role in the healing process.

Skin flap may help closure but can be affected by nonhealing skin ulcers. These ulcers are often slow healing and unresponsive to traditional treatments; consequently, the patient’s quality of life and prognosis may be severely affected [2]. To heal wounds resulting from skin transposition, local application of heterologous platelet-rich gel (PRG) has already been attempted [3], and its efficacy demonstrated in both *in vitro* and *in vivo* models [4]. The great potential of platelet-based gel has already been discussed by several authors [5–8]. Platelet-derived growth factors are involved in tissue regeneration and formation of new vessels that could improve skin flap survival [9]. The reported case is in line with the Surgical Case Report (SCARE) criteria [10].

## Case presentation

**Fig. 1:**
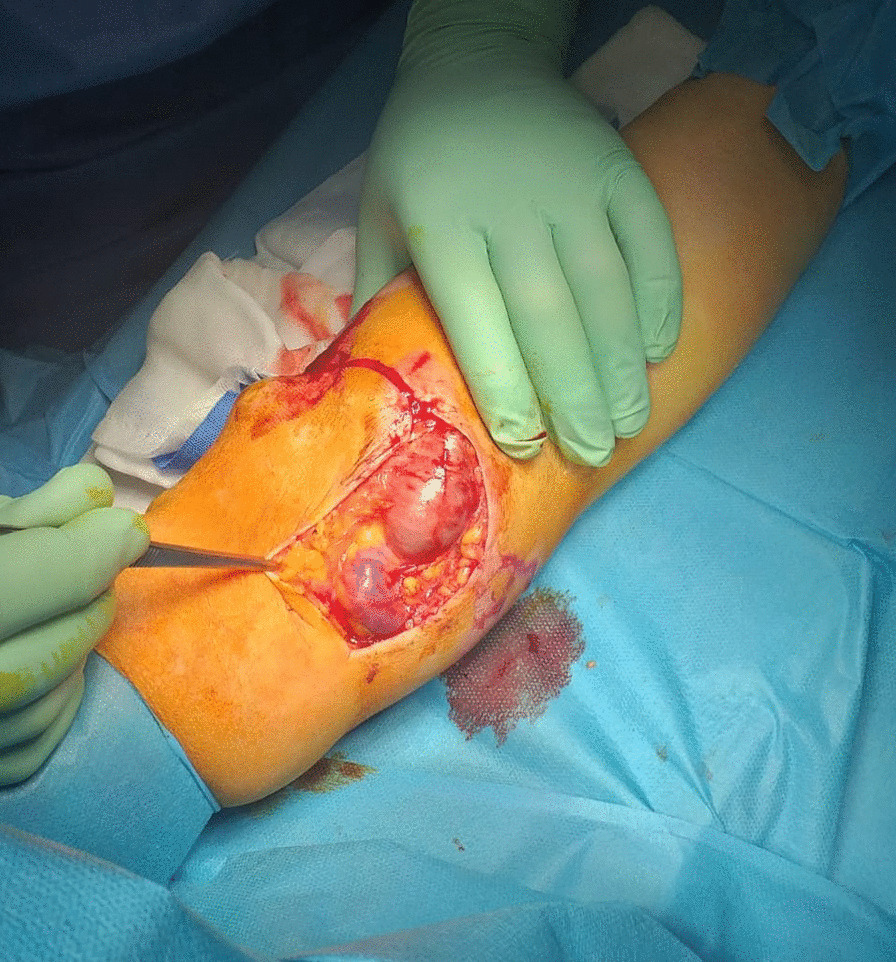
Left forearm. Intraoperative picture showing two aneurysms. The distal one is a cephalic vein aneurysm measuring 5 cm in length and 3 cm in transverse diameter; the proximal aneurysm represents the basilic vein aneurysmatic dilation (3 × 2 cm^2^)

**Fig. 2:**
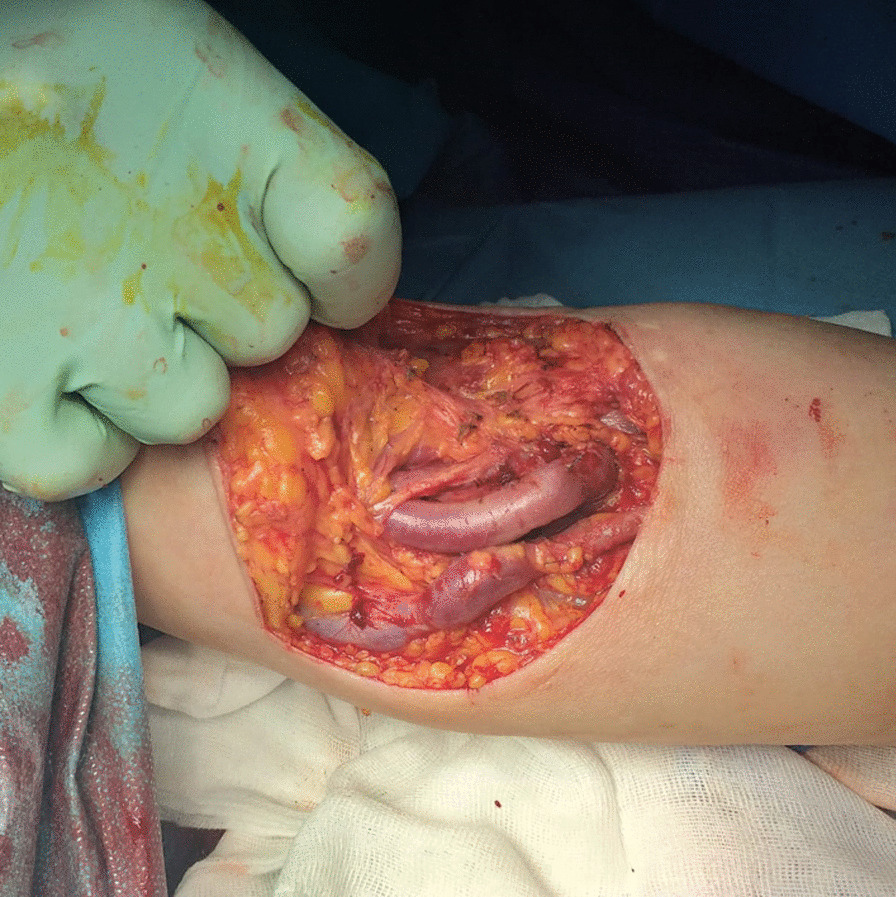
Left forearm. Intraoperative picture of the postoperative result. After AVF closure and cephalic vein aneurysmectomy, the breach of the artery wall was closed with a 5-0 polypropylene suture. Basilic vein aneurysm was then excised, and the two vascular stumps were approached with a 5-0 polypropylene suture

**Fig. 3:**
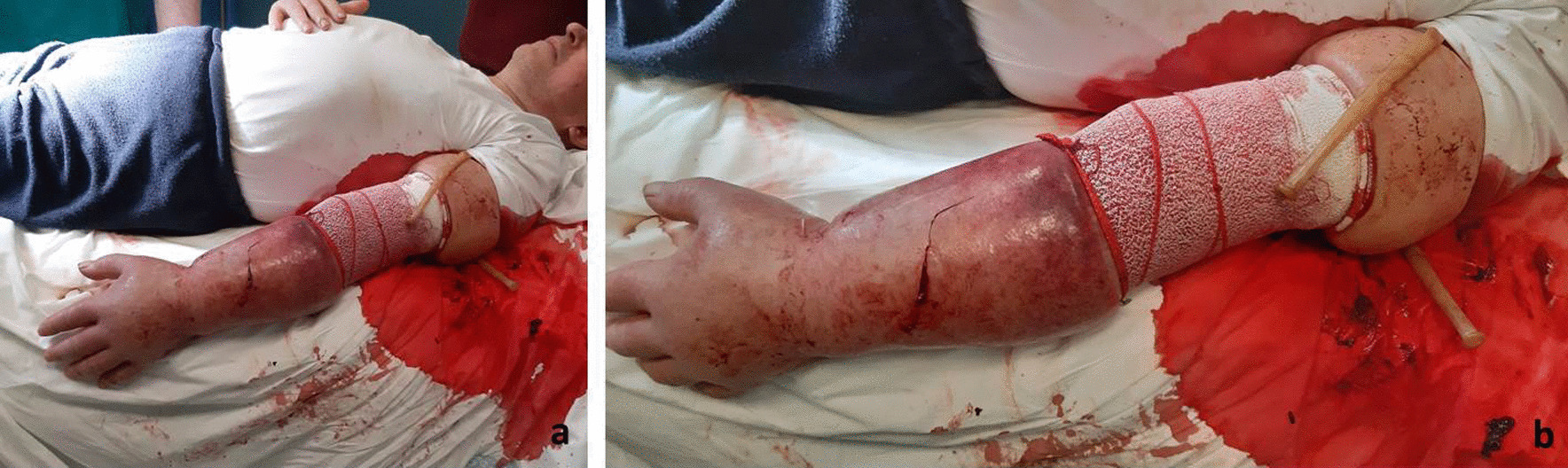
Left upper limb, 20 days after aneurysmectomy. The left forearm was edematous, and the perilesional skin showed signs of inflammation. The wound was not healed, and substance loss measured about 10 cm in transverse diameter. Blood loss was stopped by means of a tourniquet

**Fig. 4:**
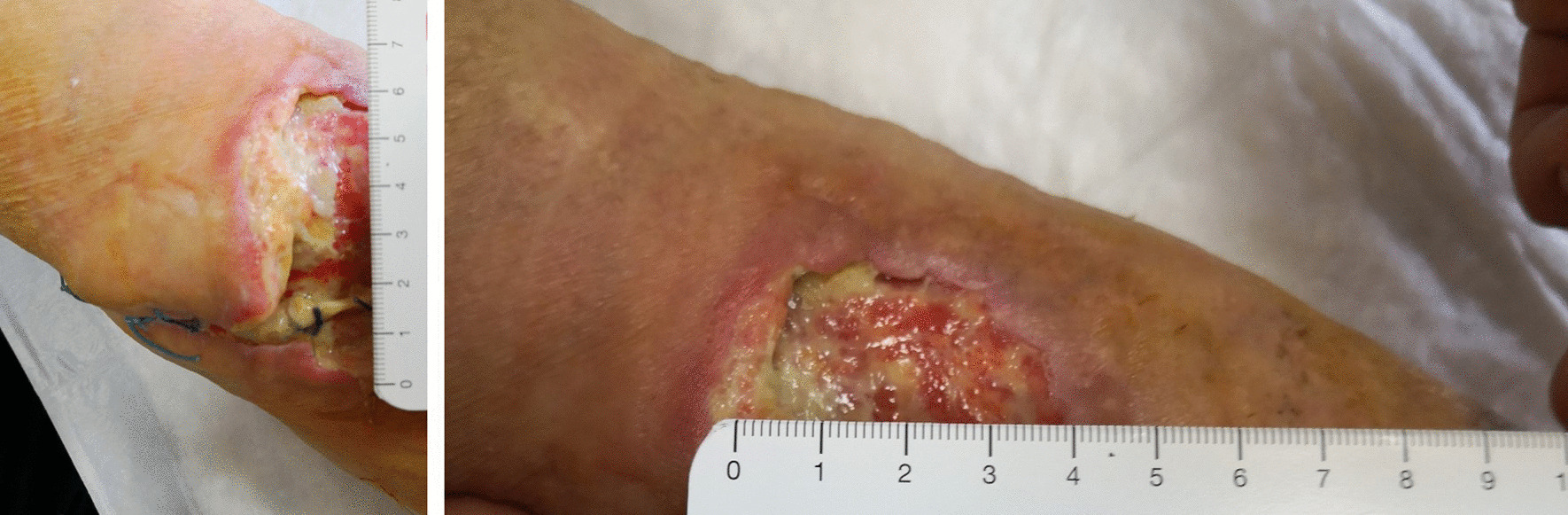
Left forearm. Surgical site soon after curettage and radial artery ligation. Loss of substance measures 6 × 4 cm^2^

A 61-year-old Caucasian male patient came to our department complaining of two large venous aneurysms on his left cephalic and basilic veins. The patient suffered from end-stage renal disease and underwent right kidney transplantation and brachiocephalic arteriovenous fistula (AVF) creation, 10 and 13 years before admission, respectively. The patient was poorly collaborative due to his cognitive retardation, likely linkable to an improperly treated meningitis during childhood. He was also affected by hypertension and colonic diverticulosis. Five years before admission, deep vein thrombosis of the upper and lower limbs was reported. Interestingly, the patient followed an immunosuppressive therapy with everolimus, prednisone, and mycophenolate mofetil. He also took atenolol, ramipril, and amlodipin to control blood pressure, and clopidogrel with subcutaneous enoxaparin in relation to his medical history of hypercoagulation. Physical examination showed a 6 × 4 cm^2^ swelling of the proximal left forearm, with no alterations of the covering skin. Thrill was present at palpation. Auscultation revealed VI/VI Levine systolic–diastolic bruit. Before intervention, informed consent was obtained from the patient’s legal representative and, only subsequently, was he premedicated with intravenous cephazoline 1 g, administered one hour before skin incision. Surgery was performed under local anesthesia by means of mepivacaine 2%. A 10-cm incision was necessary to expose the fistula and the two outflow vein dilations. Cephalic vein aneurysm measured 5 cm in length and 3 cm in transverse diameter, whereas the basilic vein aneurysmatic dilation was 3 cm long and 2 cm wide (Fig. 1; Additional file 1: Video 1). After AVF closure and cephalic vein aneurysmectomy, the breach of the artery wall was closed with a 5-0 polypropylene suture. Basilic vein aneurysm was then excised, and the two vascular stumps were approached with a 5-0 polypropylene suture (Fig. 2; Additional file 2: Video 2). The patient was discharged after two days without complications. Twenty days later, the patient came back to our attention with substantial blood loss from the surgical site (Fig. 3). The left forearm was edematous, and the perilesional skin showed signs of inflammation. The wound was not healed, and substance loss measured about 10 cm in transverse diameter. Blood loss was stopped by means of a tourniquet; intravenous administration of ceftriaxone 2 g was carried out, and the patient was immediately conducted to the operating room. Intraoperative examination revealed purulent fluid collection at the surgical site and a huge perivascular clot. Artery appeared inflamed and avascular. Debridement of the site caused substantial blood loss from proximal radial artery, close to the brachial artery bifurcation. After a failed attempt at vascular wall suture, considering the persistent infection of the surgical site (and the subsequent impossibility to use a vascular graft) and testing the patency of the ulnar artery, the radial artery was tied and completely closed. The area was cleared and, due to retracted skin margins, second-intention healing was chosen to restore normal local conditions (Fig. 4). The surgical site was treated with povidone iodine and covered with sterile gauzes. Daily medications were carried out for the next 2 weeks. After surgery, the patient showed low hemoglobin blood levels, requiring blood transfusion. To favor healing, 14 days later, 10 mL homologous (due to constantly low blood hemoglobin levels) platelet concentrate gel was applied. After application, wound was covered with a nonadherent dressing. Before the procedure, wound swab was performed. Methicillin-resistant *Staphylococcus aureus* (MRSA) colonies were detected, but no specific antibiotic therapy was established.

To prepare platelet concentrate gel, a unit of apheresis platelets from a type 0 periodic donor was used. The unit was aliquoted into 10 minibags of 20 mL each, and stored at a controlled temperature of –40 °C. Whenever a medication was performed, one minibag was thawed, divided into 10 dry tubes, and centrifuged at high speed (3500 rpm) for 5 minutes to obtain a platelet pellet stratified at the bottom of each tube. Once the supernatant plasma (rich in anticoagulant) was removed, the pellet was suspended in 10 cc of type AB virus-inactivated plasma (universal plasma donor). To allow gelling of the platelet concentrate, the Plateltex Act^®^ kit was used. The full preparation of PRP gel took around 30 min.

Medications were performed once a week (as successfully attempted in other work [1]), for 7 weeks. Before applying the PRG, the wound was prepared by surgical debridement as required to remove any dead tissue and hyperkeratotic skin, then cleaned each time with saline solution. After application, the wound was covered with nonadherent antiseptic dressing. A few layers of sterile gauze and noncompressible bandages were positioned on the region of interest. Clinical evaluation of the outcome of treatment included assessment of the size of the ulcer and degree of wound healing. Percentage of reduction was calculated using the following equation: (wound dimensions before treatment − wound dimensions after treatment)/wound dimensions before treatment × 100.

**Fig. 5:**
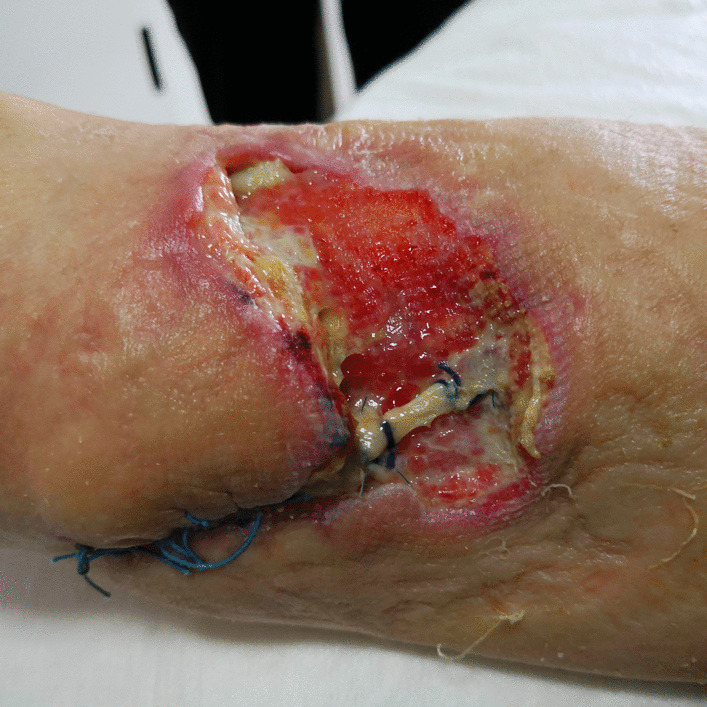
Left forearm. Skin wound 1 week after first treatment with PRG. First granulation spots appeared

**Fig. 6:**
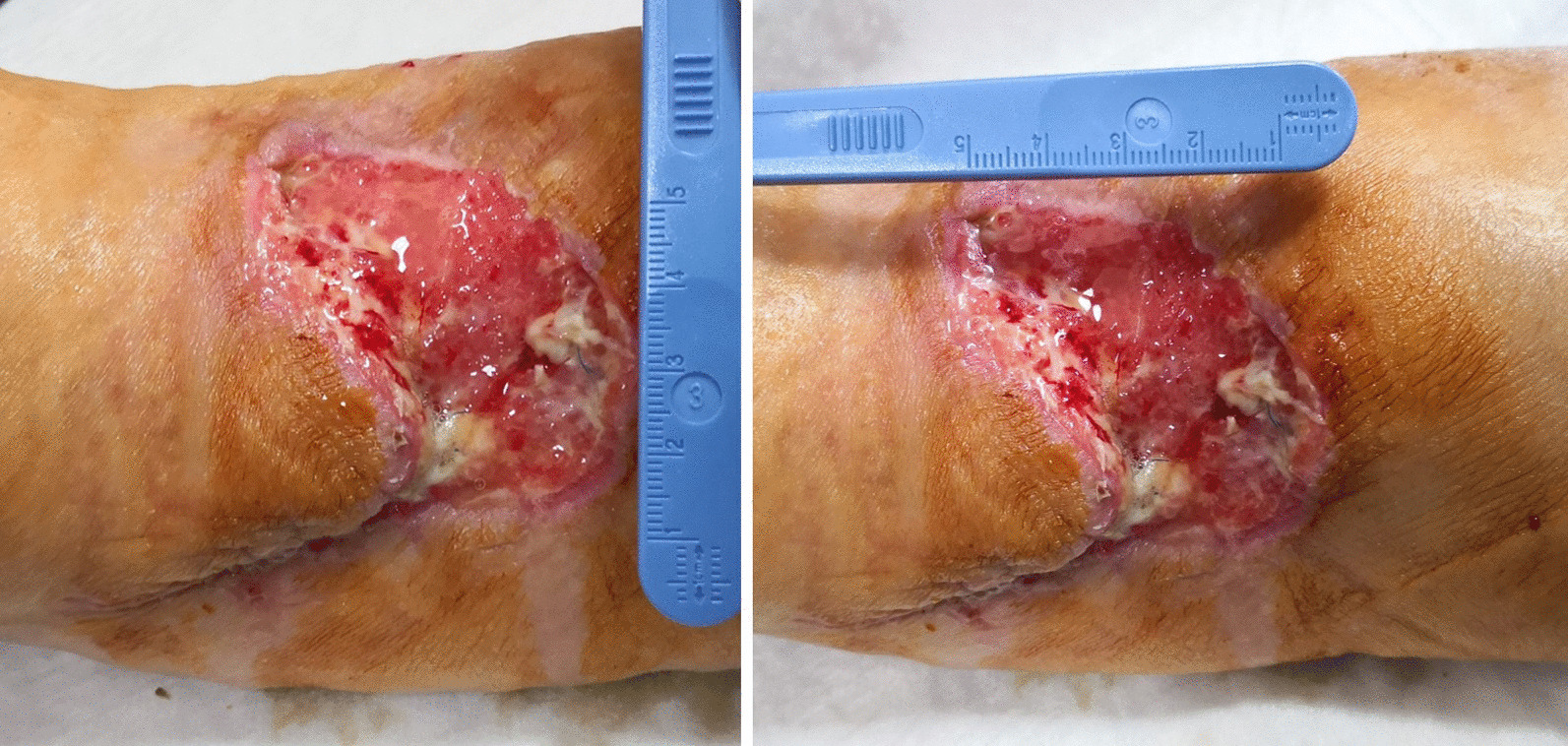
Left forearm. Skin wound at second control, before second PRG application. Loss of substance measures 5 × 3 cm^2^

**Fig. 7:**
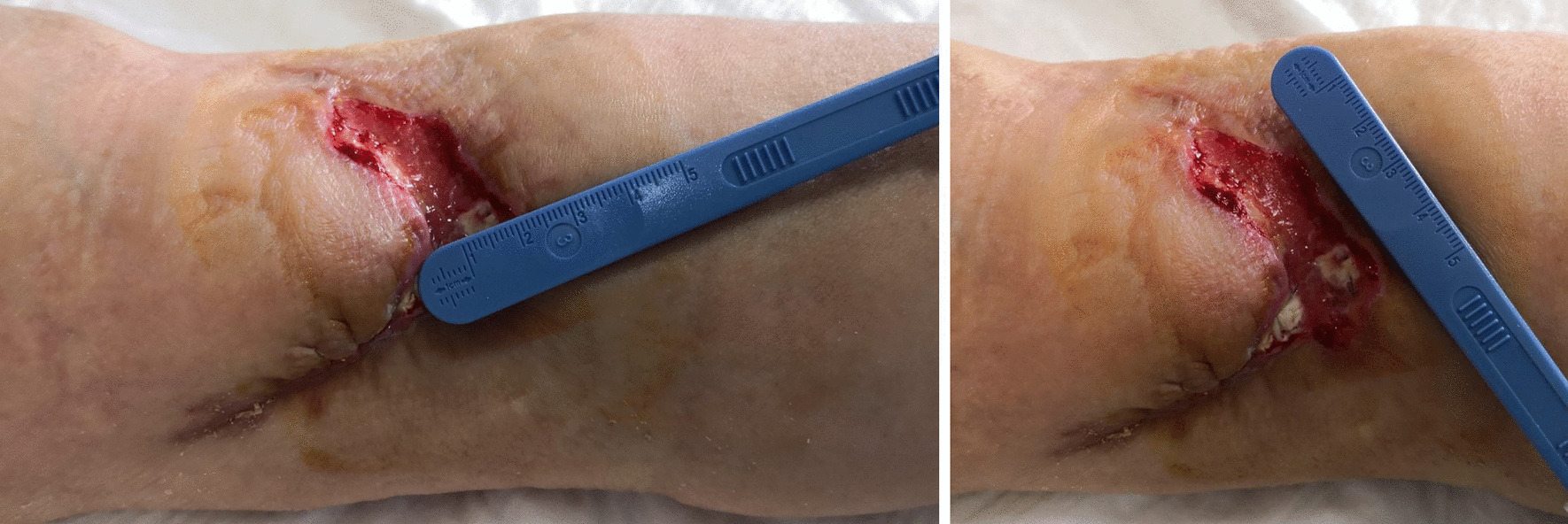
Left forearm skin wound. At third control (before third PRP application), the loss of substance measured 4.5 cm in transverse diameter and was 1.5 wide

**Fig. 8:**
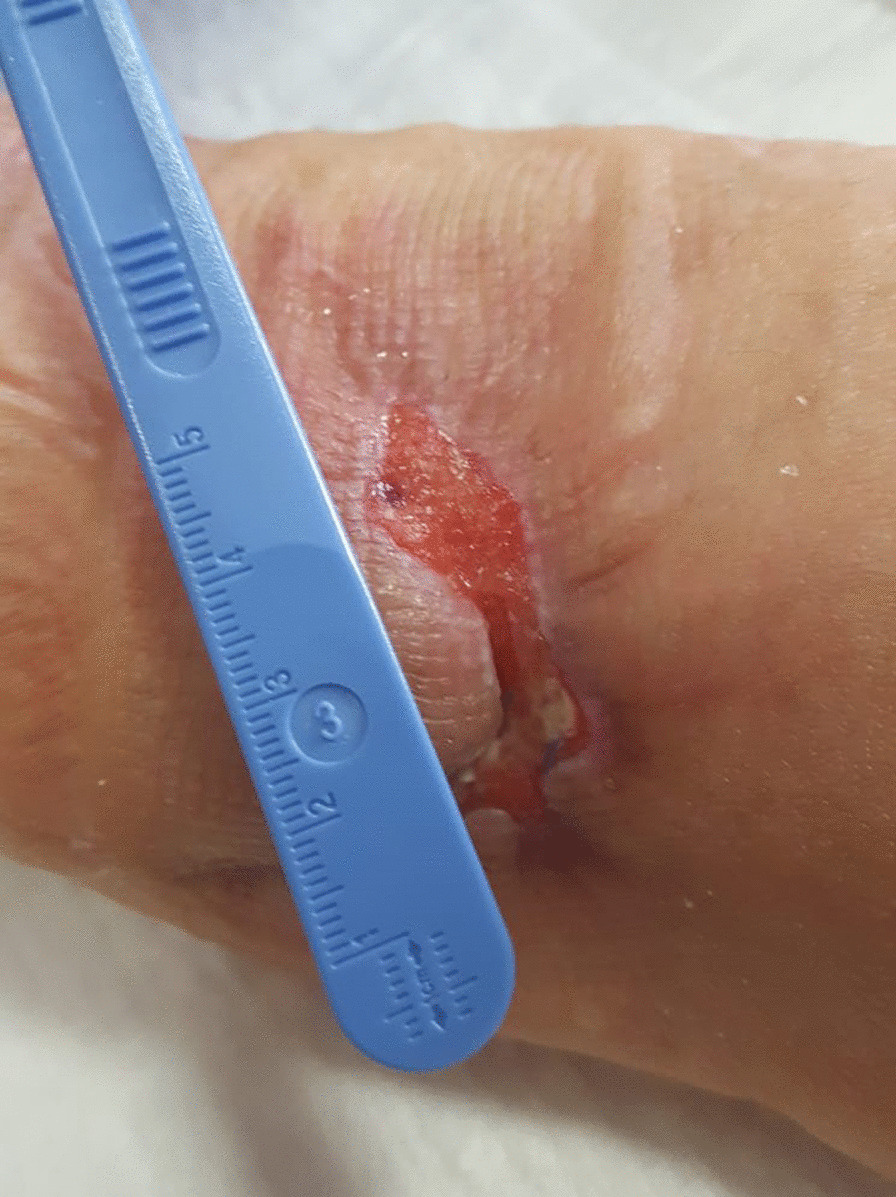
Left forearm. Skin wound 9 weeks after surgery. The loss of substance was 3.5 × 0.7 cm^2^. No signs of bacterial infection are present

**Fig. 9:**
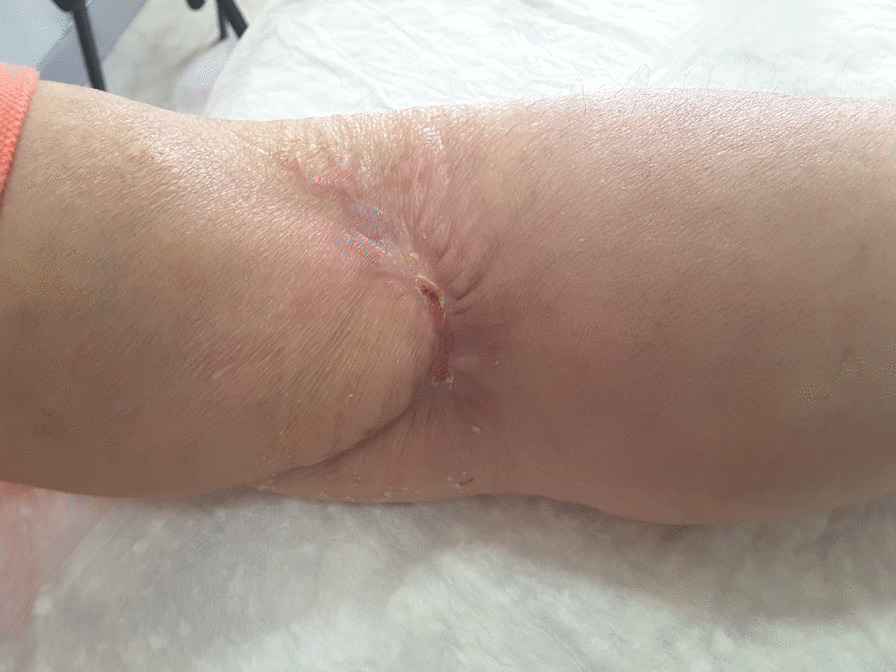
Left forearm 2 months after first application of PRG and 10 weeks after surgery. Complete recovery of the site is showed

After only seven applications of homologous platelet concentrate gel, wound completely recovered and no invasive surgical procedures were necessary. One week after the first application, the first granulation spots appeared (Fig. 5). At the second week, the wound measured 5 cm in transverse diameter and was 3 cm wide (Fig. 6). One week later, wound debridement showed that the granulation tissue covered the whole loss of substance (Fig. 7) and wound edges appeared narrower (4.5 × 1.5 cm^2^). Five weeks after the first PRG application, the wound measured 3.5 × 0.7 cm^2^ (Fig. 8). After 2 months, the wound was completely recovered (Fig. 9). There were no complications recorded in the study (Additional Files 1 and 2).

## Discussion and conclusions

Satisfactory results have also been achieved on uremic and diabetic patients suffering from diabetic ulcers, with ulcer healing and a reduction of disease-associated morbidity and decreased healthcare costs [11]. Our experience confirmed the utility of PRG for wound healing, particularly in a patient suffering from systemic sclerosis [1].

Several studies on the clinical use of PRP have already been conducted. Most of the literature ranges from dental medicine [12] to maxillofacial surgery [13], from dermatology and esthetic medicine [14, 15], to orthopedics and sports medicine [16], and even neurology [17]. PRP is also a promising therapeutic tool for the treatment of chronic mucositis [18] and for diabetic foot and leg ulcers [19]. In addition, PRP not only enhances the rate of wound healing but also reduces the neurological and neuropathic pain associated with injuries [20, 21]. Use of autologous platelet concentrates accelerates healing in dental implant surgery, orthopedic surgery, muscle and tendon repair, skin ulcers, hole repair in eye surgery, and cardiac surgery [22]. All these therapeutic properties are possible because platelet blood products are rich in growth factors and cytokines that stimulate and accelerate the wound healing process. In their literature review, Zamani and colleagues well described all the biological properties of PRG and its clinical application [6]. Platelets have great potential to act in the three different processes involved in wound healing: inflammation, cell proliferation, and extracellular matrix remodeling. The content of their granules (neurotransmitters, enzymes, cytokines, growth factors, and chemokines) stimulates wound reparation, increasing reepithelialization, contraction, and neovascularization. Platelets alone contain whatever is necessary for wound repair, and PRG guarantees the right amount of chemical signals to favor tissue regeneration. Furthermore, the presence of several enzymes (including lysozyme) exerts effective antimicrobial action [23].

Importantly, the preparation of PRG is cost-effective and relatively time-saving [24]. Furthermore, PRG is relatively safe, with few side effects or complications, mainly related to hypersensitivity [25, 26].

A randomized clinical trial conducted by Elsaid *et al*. assessed the role of PRP gel on clean nonhealing diabetic foot ulcer. Those authors reported that 25% of treated patients achieved complete healing versus none of the control group (regular dressing with saline). In total, only 8.3% of the patients treated with PRG did not show any response to treatment [27]. Another recent randomized controlled trial by Raynis *et al*. compared the effectiveness of autologous PRP gel in the treatment of hard-to-heal leg ulcers with existing conventional treatment, revealing that 25.71% of the autologous PRP group and 17.64% of control group had ulcers completely reepithelialized (*p* > 0.05). Wound size reduction was 52.35% in the autologous PRP group and 33.36% in the control group (*p* = 0.003). The autologous PRP group showed superiority over conventional treatment in wound bed coverage with granulation (*p* = 0.001) [28]. In the presented case, complete wound healing was achieved after only 6 weeks.

The percentage of reduction in the horizontal and longitudinal dimensions of the wound was 100%. This may reflect the growth-promoting effect of PRP gel.

Interestingly, despite the recorded presence of MRSA, use of PRG allowed to control surgical-site bacterial colonization without antibiotic therapy, as already reported by several authors [29, 30].

Complicated skin wounds represent an increasingly hard challenge. To date, diabetes and long-standing diseases have implied an aggressive surgical approach in most cases, with large curettages or even amputations. Regenerative medicine has offered several alternatives to invasive approaches, but is generally experimental and often very expensive. So far, PRP gel has been employed as a possible remedy to loss of substance in several branches of medicine. Unfortunately, experiences are limited and there is not extensive clinical use. With this work, the effectiveness of platelet concentrate gel in repairing challenging skin loss of substance is underlined once more. Importantly, this is one of the few cases of a large skin wound (about 10 cm in maximum diameter) treated with weekly application of PRP gel, without skin graft covering. Restitutio ad integrum was complete and autonomous (second intention) in less than 2 months, without abnormal scarring outcomes. Noninvasive procedures should always be chosen, especially in cases like the presented one in which the patient is not totally compliant. Importantly, the procedure was cost-effective and easy to perform. Furthermore, the antimicrobial properties of PRP against various microorganisms enable the potential use of PRP gel as an alternative to conventional antibiotics.

## Supplementary Information

**Additional file 1.** Left forearm. Intraoperative video showing an aneurysm of the cephalic vein (upper) and an aneurysm of the the basilic vein (lower).

**Additional file 2.** Left forearm. Intraoperative video of the postoperative result. After AVF closure and cephalic vein aneurysmectomy, the breach of the artery wall was closed with a 5-0 polypropylene suture. Basilic vein aneurysm was then excised and the two vascular stumps were approached with a 5-0 polypropylene suture.

## Data Availability

All data generated or analyzed during this study are included in this published article.

## Abbreviations

PRP: Platelet-rich plasma
PRG: Platelet-rich gel
SCARE: Surgical case report
AVF: Arteriovenous fistula
MRSA: Methicillin-resistant *Staphylococcus aureus*
